# Medical Observation.—Diphtheria

**Published:** 1861-01

**Authors:** 


### Art. II.—MEDICAL OBSERVATION.—DIPHTHERIA.

THERE are few of us who do not possess a friend with whom spectacles are a necessity. There are few probably, who, while indulging in a quiet morning gossip at the fireside with such a friend, have not witnessed a scene somewhat after this fashion: Suddenly he has started from his seat and rummaged here and there among the papers and books on the table, then he has peered intently behind and between the ornaments on the mantelpiece. Anon, the face mantling with irritation, he has darted hither and thither in the room, now tossing aside the odds and ends heaped upon this piece of furniture, now the articles piled upon that. Finally, hastening to the bell-pull he has rung violently, and almost before John Thomas or Mary Jane, as the case might be, has entered the room, he has exclaimed, "Go quickly to my dressing-room, and see if I have not left my spectacles there—" "Spectacles!" we have cried, "spectacles! why, my dear fellow, they are upon your nose!"

Now, with all due respect, we think that, as concerns diphtheria, the profession has been guilty of a fit of absence of mind not unlike that which was manifested by our friend with the spectacles in the little scene we have just depicted.

"Diphtheria," writes Mr. Simon in his second and comparatively recent Report on Public Health to the Privy Council, "had been prevalent in parts of England two years before there was any public knowledge of the fact; and under these circumstances, it has scarcely been possible to procure any trustworthy record of the first beginnings of the disease." In truth, it was towards the end of 1857 when it became generally known that a most fatal affection, of a character unfamiliar to the majority of English medical practitioners, had made its appearance in the kingdom, and had assumed an epidemic form. At the first sound of alarm the profession sought here and there, and ransacked our own and foreign medical literature, for such light as could be thrown upon the nature and characteristics of the deadly visitor. The medical literature of France, in which country the affection had long been known, was in an especial manner scrutinized, and in a short time a mass of important information was gathered together. But, unfortunately, while thus engaged in casting about for the much desired knowledge, we had almost entirely forgotten to note all those little items connected with the first appearance, and the subsequent development of the disease, which were occurring under our immediate observation, and as a consequence of this oversight (to use the mildest term) now that it has been thought proper to try to recover the early history of the epidemic, we are compelled to admit that it is scarcely possible to procure any trustworthy record of the first beginnings of the disease.

The profession, indeed, in the first excitement caused by the outbreak of a formidable malady, new to the greater portion of medical men of the present generation, forgot the true spectacles which ought to be, and it is to be presumed are, upon every medical man's nose, and there being no one standing by, as in the case of our friend, to rouse us at the earliest opportunity from our forgetfulness, we did not discover our error until we attempted to fit upon the nose another pair of glasses, rummaged out after no small amount of trouble.

It is not to be denied that since the full development of the epidemic much valuable information has been gathered respecting diphtheria, as witness more especially the reports of Drs. Greenhow and Sanderson, printed in Mr. Simon's report. Our quarrel rests with the fact of the absence of reliable information respecting the early development of the epidemic. This defect is all the more vexatious, and the lesson to be learnt from it ought to be all the plainer, because such an admirable opportunity has not been afforded to the medical men of this country, for the scientific study of the origin of an epidemic, and for putting our knowledge of the laws of epidemics on a sounder foundation, since the first outbreak of cholera in 1832.

Thus stand the facts, that, since 1855, a deadly epidemic of a well-marked, but with us of the present day, in this country, novel disease, has broken out among us, has swept over the greater part of the kingdom, is still extending its ravages, and is apparently going to become domiciled among us, and yet we can give no account of the beginning of this disease. Nay more, in discussing that beginning, and also that fundamental problem of epidemiology, the development of epidemics, we have not essentially advanced one step beyond the theories entertained eighteen centuries ago. Of diphtheria, as of cholera, we question whether the disease was brought among us by contagion from without, or whether it was developed solely by causes acting within, the kingdom. And so we find Philo,* the physician, asserting that in his day tubercular leprosy was a comparatively new disease in Italy; and Pliny the elder† maintaining that the affection had been conveyed into Italy by the troops of Pompeius Magnus, when that general returned from Asia; while Athenodorus,‡ the philosopher, contends against Philo, that the disease had long existed in Italy, and that in their days it had solely become more prevalent, increasing also in virulence. Could anything show more clearly the existence of a chronic and fundamental defect in medical observation than the persistence at the present time, in every essential respect, of the same vague pseudo-scientific theories concerning the origin of epidemic diseases which existed in the first century?

If we would remedy the deficiency here adverted to, it is requisite first of all that we should endeavour to ascertain the causes which give rise to it. Now, it is evident that the power of removing the evil will be in proportion to our comprehension of its nature. This, we think, may be best obtained by an examination of the two chief inquiries which have been instituted into the reigning epidemic, the one under the authority of the Privy Council, the other by the Council of the Epidemiological Society.

The first-named inquiry was conducted (after the usual mode of Privy Council inquiries concerning epidemic disease) by highly qualified inspectors sent from London to the principal seats of the disease, and by correspondence with infected districts. The inquiry was confined solely to infected districts, and was instituted in such localities only after the disease had unmistakeably broken out in them. It is obvious that an inquiry thus carried

---

* *Plutarch, Sympos.* B. viii. c. 9. † *Nat. Hist.*, B. xxvi. c. 5.

‡ *Plutarch, Sympos.* B. viii. c. 9.

out, however valuable and requisite in many respects, must, in all that relates to the origination and development of the epidemic, be entirely dependent upon the habits of observation possessed by local observers. These being defective, we should expect to read "it has been scarcely possible to procure trustworthy records of the first beginnings of the disease." Again, the absence of all comparative observation, that is to say of observation carried out in uninfected districts as well as infected, or of information on the progress and characteristics of allied affections over a series of months or years preceding the inquiry, at once destroys the power of scientific appreciation of the facts ascertained bearing upon the spread of the epidemic. In truth, the Privy Council method of inquiry, can at the best render but a partial account of an epidemic, and however important this may be, in reference to an immediate and practical dealing with the ravages of the disease (no doubt the great object of the inquiry), it can never supply what we contend should be the great aim of all such inquiries (since the strictly scientific must include the strictly practical), a scientific history of the rise, progress, or development of an epidemic in the kingdom, or indeed, in any one district of it.

The Epidemiological Society conducted its inquiry in another fashion. Working by a committee, it distributed a circular among its members and others asking for information on diphtheria, and drawn up so as to secure precision and uniformity in reports on the disease. The Report of the Committee, recently published,* gives a very instructive account of the manner in which this circular was responded to. The Committee state that "Although (apart from publication in the medical journals) upwards of two hundred circulars, asking for information, and containing the foregoing suggestions, were distributed in various parts, over almost the entire surface of the kingdom, and notwithstanding that, several weeks after the distribution, the attention of many non-resident members of the Society was particularly directed, by a brief note, to the importance of giving the information sought in the circular, the Committee regret to have to state, that during the ten months that the inquiry has been kept open, they have received only *thirteen* specific reports on diphtheria, and *twenty-two* replies, in which the writers state, in very general terms, either the entire absence of diphtheria from their localities, or a lack of personal, or a very slight acquaintance with the disease."

Again, in concluding their Report the Committee state:—

"That although the information obtained by them in the present inquiry is exceedingly imperfect, yet it is sufficient to show that if the inquiry had been supported by the members of

---

* *Transactions of the Epidemiological Society of London*, vol. i. part 1, 1860.

the Society to the extent that the committee had hoped it would have been, a large amount of most valuable information respecting diphtheria would have been obtained—information of a character that can be obtained in no other manner than by the systematic co-operation of many and widely separated observers to one and the same end. The committee would suggest that the Society should take into consideration the propriety of adopting other and additional means (if such can be devised) for promoting or insuring a more satisfactory co-operation of its members in such inquiries as the Society may set on foot; for the committee feel assured that the present inquiry has proved less successful than might have been anticipated, not so much from indisposition of the members to aid, as from an erroneous estimate of the value of the information which they possessed relative to the subject of inquiry. Thus in the majority of letters with which the committee have been favoured in answer to their circular, the writers have contented themselves simply with stating that diphtheria has not appeared in their neighbourhood, or that they have seen but one or two cases of the disease, and that consequently their experience would be of no value to the committee, notwithstanding that the committee had specifically asked for particular information respecting the prevailing character of throat affections where the disease had not manifested itself, and for many items of information where it had, even if it were but in a solitary instance."

These observations of the Diphtheria Committee of the Epidemiological Society, showing the extent and indicating the sources of the failure of the inquiry conducted by it, are of great interest. What was to be deduced from the result of the Privy Council inquiry, is here made more clearly apparent, to wit, the absence of any methodic system of epidemiological observation among the medical men of the kingdom, and the results of both inquiries most conclusively show that without this preliminary observation all general inquiries whatever into the nature and development of epidemics must of necessity prove more or less abortive. The local inquiries instituted by the Privy Council, in infected localities, and carried on by men accustomed to medical researches, lead to the accumulation and verification of a vast amount of information of unusual value and essential to a knowledge of epidemics. The names of the inspectors, and above all of the distinguished medical officer of the Privy Council, Mr. Simon, are a sufficient guarantee for this being the case. The actual results of the inquiry made by the Epidemiological Society, although meagre, are by no means wanting in value, and particularly in suggestiveness; but neither the method of inquiry adopted by the medical officer of the Privy Council nor that made use of by the

Epidemiological Society, can be looked upon as meeting the requirements of epidemiological observation at the present time. In fact, we possess no system of epidemiological observation which can be legitimately termed scientific, and for the lack of this the greatest opportunity for accurately noting the origin, the rise, and the progress of a great epidemic which has ever been presented to the medical profession of any country under the sun, has been almost utterly lost to us. It would be foolish to waste time in speculating what might have happened had we been enabled to observe accurately the epidemic from its earliest foreshadowings, but truly one cannot prevent some feelings of vexation when, knowing how greatly our knowledge of the predisposing causes of epidemics has been developed of late years, we remember that in the early history of outbreaks such as that of diphtheria, we can alone hope to obtain an explanation of the incongruities and anomalies which beset our knowledge of the exciting causes of epidemics, as well as a sounder acquaintance with the etiology of these outbreaks in general. The mischief, however, being done, it remains for us to set ourselves heartily to work to prevent the recurrence of such an evil. The lesson should be a bitter one to those who will read it rightly; let us try to make it an useful one.

We hold that the lesson clearly teaches us that the great defect with which we have to contend in this matter is the absence of a true system of epidemiological observation. This being admitted, the question follows, how is this evil to be remedied?

At the first glance it might seem that an authoritative Registration of Disease would solve the question and supply the method sought. This we think is an error. A Registration of Disease is undoubtedly a necessary element in a thorough method of observation, and consequently it is incumbent upon the profession, on this account, as also on account of the immediate advantages that would arise to the nation out of such a registration, to aid heart and soul those men who are now struggling to secure so great an addition to the statistical records of the kingdom. But while a registration of disease would give us positive information as to the existence, the amount, the variations in time and place, and the distribution of different diseases in the country, it would be silent, however well conducted, and almost of necessity, regarding the development, the mutations in form, or (assuming such a thing) the transformations of disease. A registration of disease, together with the records of mortality, would furnish us with the positive facts of disease in a given locality, district, or country, and would provide us with the most accurate basis for the study of the etiology of any given disease or class of diseases, but it would not, and, indeed, could not, supply that accurate study of the phases of disease, and of the alliances of diseases, upon which the unravelment of the laws of etiology must ultimately depend.

We hold then that, even if, as is to be hoped, a registration of disease be shortly obtained, the defects of existing methods of observing epidemics will in a great measure remain, and these we believe can only be got rid of by the formation of a corps of competent observers, banded together for one and the same object, and working steadily upon one and the same system.

If we reflect upon the subject, few things are more remarkable than that, with the large number of highly qualified scientific medical observers which so markedly characterizes the medical profession of this country, there should be so entire an absence of any combined system of observation, not only on epidemiological questions, but on all questions connected with the prevalence or spread of disease. But the fact of the existence of numerous individual observers scattered over the kingdom, would indicate that the formation of a corps working together to one end is a feasible project. Moreover, the experiment has in part been tried. For when Dr. Richardson established the *Sanitary Review*, he projected a scheme for the registration of certain facts concerning epidemics in the pages of that journal, and contributors at once and willingly tendered their services from all parts of the kingdom.

The method of observation would prove a much more difficult matter to solve, for, dealing chiefly with the most obscure questions of epidemiology, it must, in the first instance, be of necessity tentative. But even here, the recent investigations into diphtheria yield us considerable light, and plainly indicate the chief points at which a method should aim. It should be—

1. Directive of observation.

2. Calculated to foster habits of combined observation among the profession at large.

3. It (and most emphatically we would say this), should be conceived in such a manner as to react upon our schools of medicine, so that the habits spoken of may, in the first instance, be developed, and the system of observation determined upon learned, there.

That is to say, the method should be calculated for the future as well as the present, we of this generation not merely seeking to widen the foundations of knowledge for the next, but also endeavouring especially to train up the nascent representatives of that generation in those habits of observation which we ourselves mostly fail in.

The London Medical Society of Observation did good service by the publication of its manual on *What to Observe at the Bed-*

*side and after Death in Medical Cases;* and Dr. Ogle has also done good service by the recent publication of a paper on the *Clinical Observation of Diphtheria,* in Beale's *Archives of Medicine.* The work of the Medical Society of Observation must be looked upon as having fostered more accurate habits of clinical observation; and it is to be hoped that Dr. Ogle's paper will induce a more systematic observation of cases of diphtheria. Both works are indeed highly valuable contributions to a better system of medical observation, but neither meets the defects we have pointed out. Can these defects be remedied in the fashion we have indicated? We think that they may, and that, moreover, that the Epidemiological Society is the machinery by which the reform may be best carried out. If the Society would grapple with the suggestion of its Diphtheria Committee, that is to say, "take into consideration the propriety of adopting other and additional means (if such can be devised) for promoting or insuring a more satisfactory co-operation of its members," we feel little doubt that it would soon get to the core of the matter.

---

